# *In Vitro* Antibacterial, DPPH Radical Scavenging Activities, and *In Silico* Molecular Modeling of Isolated Compounds from the Roots of *Clematis hirsuta*

**DOI:** 10.1155/2024/3152929

**Published:** 2024-03-25

**Authors:** Tolessa Duguma, Yadessa Melaku, Ankita Garg, Urgessa Ensermu

**Affiliations:** ^1^Department of Applied Chemistry, Adama Science and Technology University, Adama, Ethiopia; ^2^Department of Applied Biology, Adama Science and Technology University, Adama, Ethiopia

## Abstract

*Clematis hirsuta* is one of the traditional medicinal plants used in Ethiopia to treat different ailments, such as cancer and diseases related to the respiratory system. This study aimed to isolate the phytochemical components of the root of *C. hirsuta* and evaluate their *in vitro* and *in silico* biological activities. Oleic acid (1), palmitic acid (2), sterols (3 and 4), boehmenan (5), and carolignans E (6 and 7) were isolated by silica gel column chromatography and preparative thin layer chromatography and characterized by NMR spectroscopy. Compounds 5–7 were isolated from the plant for the first time. At 5 mg/mL, the inhibition zone of evaluated compounds ranged from 8.80 to 11.10 mm against all selected bacteria. The MIC of the MeOH and *n*-hexane: EtOAc (1 : 1) extracts was greater than or equal to 50 mg/mL against all selected bacteria. At 62.5 *μ*g/mL, the % DPPH radical scavenging activity of tested compounds ranged from 30.3% to 92.1% with an IC_50_ value of 19.4 to 2.1 *μ*g/mL. The results of molecular docking studies indicated that the docking scores of compounds 3–7 ranged from −6.4 to −7.9 kcal/mol against *E. coli* DNA gyrase B, −8.3 to −9.0 kcal/mol against the *Pseudomonas* quinolone signal A, −7.1 to −8.5 kcal/mol against pyruvate kinase M2, and −7.9 to −8.5 kcal/mol against human topoisomerase *IIβ*. The results of the *in silico* antibacterial activity of compounds **3**, **5**, and **6** supported the *in vitro* antibacterial test results. Compound **5** had a better docking score against human topoisomerase *IIβ* than the other test samples demonstrating its potential as an anticancer agent. Therefore, compounds 3–7 could be considered as a lead for developing antibacterial and anticancer drugs. Moreover, the presence of these active phytochemicals supports the traditional use of this plant against cancer and bacteria.

## 1. Introduction

*Clematis hirsuta* Perro & Guill (Ranunculaceae) is a shrub that belongs to the genus *Clematis* [1]. The plant is endemic to Cape Verde, the Sahara, tropical Africa, and the South West Arabian Peninsula. It can grow up to 4 m and contains leaves of the pinnate type with 5 leaflets. The shape of the leaves is suborbicular to ovate with tips of acuminate, acute, or subobtuse with cordate to rounded and rarely truncate at the base. The margins of the leaves are longer in the central lobe and shorter on each side. The plant contains inflorescences composed of many flowers with pedicels 1 to 3 cm long and 1 to 2.7 cm long cream or white sepals. It has a flower bud with a spherical to ellipsoid shape containing rounded to acuminate tips [1].

*C*. *hirsuta*, locally called *Hidda feetii* in Afan Oromo [2], is a plant that has traditionally been used to treat different diseases including swelling, cough, cataracts, and leishmaniasis [3–6]. The fresh leaves of the plant are also used to treat earache, cancer, and tuberculosis [7, 8]. Its roots are administered orally to treat respiratory tract problems [5]. The whole part of the plant is used for the treatment of fungal and trypanosomal diseases [9] and wounds [10]. Pharmacological reporting has shown that the various solvent extracts of the leaves and roots of *C*. *hirsuta* exhibit antibacterial activities [2, 11–13]. The MeOH extracts of the root, the petroleum ether, CHCl_3_, and EtOH extracts of the leaves were also reported to have DPPH radical scavenging activities [2, 13]. The phytochemical screening of the root of the plant revealed the presence of alkaloids, saponins, tannins, flavonoids, phenols, and glycosides [2]. The aerial part of *C*. *hirsuta* was also reported to have *β*-amyrin, lupeol, *β*-sitosterol, oleanolic acid, and stigmasterol glycoside [14].

Despite the tremendous traditional use of the plant against various life-threatening diseases, there is no prior report on the isolation and characterization of secondary metabolites from the root extracts of *C*. *hirsuta*. Furthermore, the biological activities of the root of the plant were also not well explored. Therefore, this work aimed to isolate and characterize phytochemicals from the root extracts of *C*. *hirsuta* and assess their antibacterial and 2,2-diphenyl-1-picrylhydrazyl (DPPH) radical scavenging activities. Hence, in this paper, we presented the findings on the phytochemical constituents, *in vitro* antibacterial, and DPPH radical scavenging activities of root extracts of *C*. *hirsute* for the first time. The results of the *in silico* molecular docking analysis, drug-likeness, and toxicity of isolated compounds were also included.

## 2. Material and Methods

### 2.1. Plant Material Collection, Identification, and Preparation

The root of *C*. *hirsuta* was collected in July (Summer 2021) from Waldoro village, Homa Kebele, Abbay Chomman Woreda, Horo Guduru Zone, Oromia Regional State, which is about 400 km west of the capital, Addis Ababa, Ethiopia. The plant was identified with the help of available literature and authenticated (TD05/2021) by botanist Mr. Melaku Wendaferash at the National Herbarium, Department of Biology, and Addis Ababa University, Ethiopia. Then, the collected plant material was immediately brought to the Organic Chemistry Laboratory of Adama Science and Technology University and allowed to dry in the open air at room temperature under shade. The dried plant material was then pulverized using an electric grinder and stored at 4°C.

### 2.2. Extraction and Isolation

The pulverized root of *C*. *hirsuta* (550 g) was successively extracted with *n*-hexane: EtOAc (1 : 1, 2.75 L) and MeOH (2.75 L) maceration for 72 hours. It was filtered and concentrated using a rotary evaporator at 40°C to provide 4.5 g (0.82%) and 8.5 g (1.55%), respectively. Both extracts (13 g, 2.37%) were combined. About 10 g of the extract was adsorbed on 10 g of silica gel and subjected to silica gel column chromatography (size: 80 mm internal diameter by 650 mm length with 29/32 joint type) using 170 g of silica gel. The column was eluted with a gradient of *n*-hexane: EtOAc (1 : 0 to 0 : 1) and CHCl_3_: MeOH (9.9 : 0.1 to 0 : 1). A total of 305 fractions were collected with a volume of 10 mL except fractions 1, 28, 44, 45, and 151 which were collected with a volume of 100 mL and fractions 46, 51, 72–75, 94, 100–103, 142, 161, 253-254, 296-297, and 305 which were collected with a volume of 200 mL.

Fractions 28–43 (230 mg), which were eluted using *n*-hexane: EtOAc (7 : 3), were further fractionated with the same eluent using silica gel column chromatography (size: 60 mm internal diameter by 360 mm length with 14/23 joint type) to obtain compound **1** (15 mg). Likewise, fractions 44–46 (330 mg), which were eluted with *n*-hexane: EtOAc (7 : 3), were eluted in isocratic mode using column chromatography with silica gel to obtain compound **2** (16 mg). Fractions 47–55 (165 mg), which were eluted using *n*-hexane: EtOAc (65 : 35), were fractionated in isocratic mode on silica gel column chromatography to obtain a mixture of compounds **3** and **4** (32 mg). Fractions 86–95 (330 mg) were eluted using *n*-hexane: EtOAc (1 : 9). Using the same solvent ratio, the fractions were eluted in isocratic mode on silica gel column chromatography and further purified using PTLC to obtain compound **5** (18 mg) and a mixture of compounds **6** and **7** (30 mg).

### 2.3. Evaluation of Antibacterial Activities

#### 2.3.1. Disk Diffusion Method

The evaluation of the antibacterial activity of extracts and isolated compounds was carried out using the disk diffusion method against clinical isolate bacteria, including two Gram-positive bacteria (*Staphylococcus aureus* ATCC 25923 and *Streptococcus pyogenes* ATCC 19615) and two Gram-negative bacteria (*Escherichia coli* ATCC 25922 and *Pseudomonas aeruginosa* ATCC 27853) using the standard procedure [15]. Thus, 38 g of Mueller-Hinton agar medium (MHA) was dissolved in 1 L of distilled water and heated to dissolve until completely and allowed to cool. After sterilization using an autoclave at 121°C for 15 minutes, 25 mL of the medium was dispensed into a 90 mm Petri dish to a depth of approximately 4 mm and allowed to solidify at room temperature and stored at 4°C. The test bacteria was diluted in sterile Muller-Hinton broth to adjust the turbidity to the 0.5 McFarland BaCl_2_ standard solution to get 1.5 × 10^8^ cfu/mL bacteria suspension. Within 15 min, each inoculum was inoculated in a Petri dish using a sterile swab. A 6 mm sterile disc made of Whatman filter paper No. 1 was saturated with 20 *μ*L of 100 and 50 mg/mL of each extract and with 5 and 2.5 mg/mL of each isolated compound and then placed on the surface of the Petri dish. The Petri dish was incubated at 37°C for 24 hours. The diameter (in millimeters) of the inhibition zone was measured. The extent of activity of the samples was compared to a sterile 6 mm disc saturated with 20 *μ*L (10 *μ*g) of 0.5 mg/mL ciprofloxacin. All analyses were carried out in triplicate.

#### 2.3.2. Minimum Inhibitory Concentration (MIC) of Extracts

The agar dilution method was used to evaluate the MIC of the MeOH and *n*-hexane: EtOAc (1 : 1) extracts using the procedure [16, 17] with some modifications against the selected bacteria. In detail, the MHA media was prepared as described above. A stock solution of 100 mg/mL was prepared by dissolving 400 mg of each extract in a small amount (2 mL) of DMSO. Afterwards, it was adjusted to the final volume of 4 mL using the MHA solution and serially diluted to the working solution 50, 25, 12.5, 6.25, and 3.125 mg/mL in the MHA solution. About 500 *μ*L of each concentration was distributed into 100 mL labeled sterilized beakers and mixed with 24.5 mL of MHA media solution. The mixtures were then poured into a Petri dish labeled with the respective concentration of extracts. Following that the plate was allowed to dry at room temperature. Approximately 5 *μ*L of suspension (density adjusted to 0.5 McFarland turbidity units, 1 to 2 × 10^8^ cfu/mL) was transferred as a spot on each plate. The plates were incubated at 37°C for 24 hours. The lowest concentration that completely inhibits visible growth, as judged by the naked eye, was taken as MIC. Agar plates without test extract and with standard antibiotics, ciprofloxacin (0.0625, 0.03125, and 0.015625 mg/mL), have been used as a negative and positive control, respectively.

### 2.4. Radical Scavenging Activity

The radical scavenging activity of the extract and isolated compounds was evaluated using the DPPH (1,1-diphenyl-2-picrylhydrazyl) radical according to the reported procedure [18]. In detail, 4 mg of DPPH was dissolved in 100 mL methanol to obtain 40 *μ*g/mL and stored in a dark bottle at 4°C. The crude extracts and isolated compounds were separately dissolved in methanol to prepare 1000 *μ*g/mL. Using a two-fold serial dilution in methanol, 500, 250, 125, and 62.5 *μ*g/mL working solutions were prepared. Then, 1 mL of each was mixed with 1 mL of DPPH. After 30 min of incubation in darkness at room temperature, the absorbance was taken using a UV-vis spectrometer at 517 nm adding methanol as a blank. The percent (%) of DPPH inhibited by the samples was calculated as (*A*_*c*_ − *A*_*S*_/*A*_*c*_) × 100%, where *A*_*c*_ is the absorbance of the control and *A*_*s*_ is the absorbance of the sample. IC_50_ was calculated from the regression line [19]. Ascorbic acid was used as standard. All analysis was carried out in triplicate.

### 2.5. *In Silico* Molecular Docking Analysis of the Isolated Compounds

The docking was conducted using the reported procedure [20]. Specifically, the structures of the compounds were drawn using ChemDraw 22.0 and saved in (.mol file) format before they were optimized using Discovery Studio Visualizer 21.1 software [21] and saved in (.pdb file) format. To validate the *in vitro* antibacterial activities conducted against *E*. *coli* and *P*. *aeruginosa*, two bacterial enzymes, viz. *E*. *coli* DNA gyrase B (PDB ID: 7P2M) which is essential for DNA replication in *E*. *coli and Pseudomonas* quinolone signal A, PqsA (PDB ID: 5OE4), that is responsible for *P*. *aeruginosa* pathogenicity. Similarly, to predict the anticancer activities of isolated compounds, two cancer-causing enzymes were selected. These are pyruvate kinase M2, PKM2 (PDB ID: 4G1N) that plays a vital role in cancer cell metabolism, and human topoisomerase *IIβ* (PDB ID: 3QX3) that participates in the multiplication of human cancer cells. All target enzymes were downloaded from the protein data bank and saved in (.pdb file) format. Then, using Discovery Studio Visualizer software [21], the binding sites were identified, and all ligands and water molecules were removed from the target proteins. The docking was carried out using AutoDock Vina with MGL tools 1.5.6 and Python 3.10.9 as supporting software. Each run was carried out for nine conformers. The least binding energy conformer with the lowest root mean square deviation (RMSD) was selected and analyzed using the Discovery Studio Visualizer to show the interaction of the ligand and target enzymes in the form of a 2D and 3D structure. The result was compared with the result of the standard antibacterial drug, ciprofloxacin, and the standard anticancer drug, abiraterone.

### 2.6. *In Silico* Pharmacokinetics and Toxicity of Isolated Compounds

The canonical simplified molecular input line entry system (SMILES) was taken from the PubChem database and submitted to the SwissADME online tool to estimate physicochemical (molecular weight, number of rotatable bonds, number of hydrogen bond donors, number of hydrogen bond acceptors, and total polar surface area), lipophilicity (log *P*_*O*/*W*_), and drug-likeness (Lipinski's rule) [22]. For the prediction of *in silico* acute oral toxicity, organ toxicity (hepatotoxicity), and end points of toxicity (carcinogenicity, immunotoxicity, mutagenicity, and cytotoxicity), isolated compound SMILES was submitted to the Pro Tox II online tool [23]. The result of the isolated compounds was compared with those of ciprofloxacin and abiraterone.

## 3. Results and Discussion

### 3.1. Characterization

**Figure 1:**
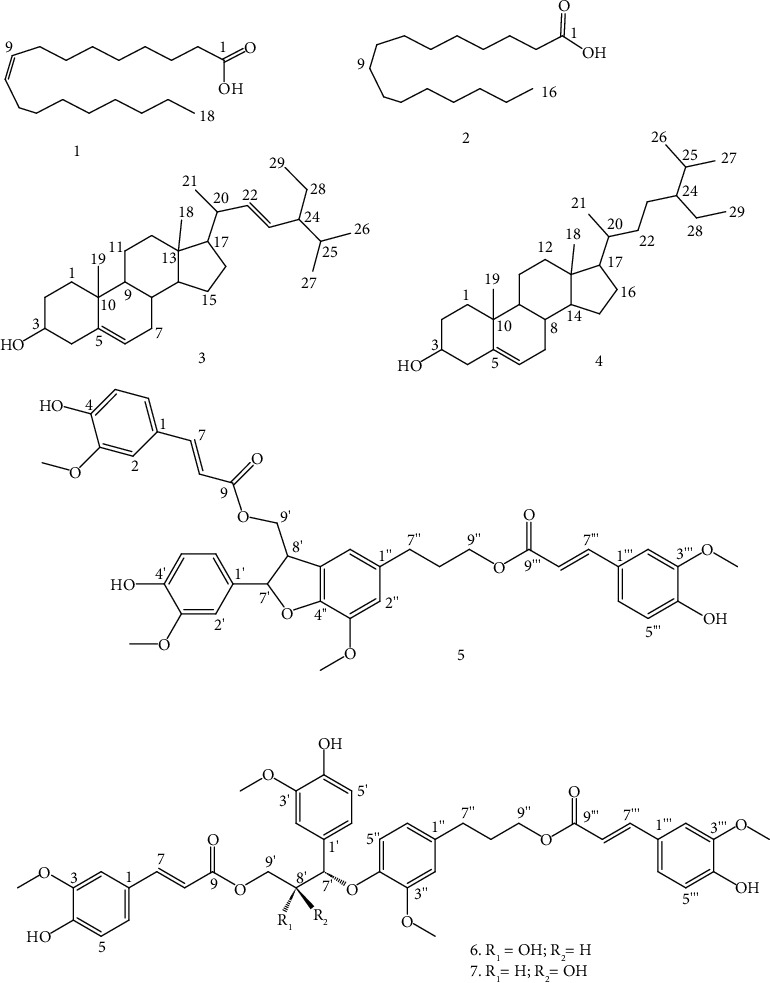
Chemical structures of isolated compounds from the roots of *C. hirsuta*.

Seven compounds (Figure 1) including oleic acid (**1**), palmitic acid (**2**), sterols (**3** and **4**), boehmenan (**5**), and carolignans E (**6** and **7**) were isolated and characterized from the root extract of *C*. *hirsuta*. The structures of the isolated compounds were elucidated using NMR spectroscopy with the details presented as follows.

Compound **1** was obtained as a pale yellowish solid with a melting point of 13–15°C. TLC showed a spot at *R*_*f*_ value 0.6 using *n*-hexane: EtOAc (9 : 1) as a mobile phase. The ^1^H-NMR (400 MHz, CDCl_3_) spectrum (Figure S1) indicated the presence of 33 protons, including methyl protons at *δ* 0.90 (3H, *t*, *J* = 6.5 Hz, H-18), ten methylene protons at *δ* 1.30 (20H, br. s, H-4-H-7, and H-12-H-17), four additional methylene protons at *δ* 1.65 (2H, m, H-3), 2.04 (4H, br. s, H-8 and H-11), and 2.37 (2H, *t*, *J* = 6.4 Hz, H-2) and olefinic protons at *δ* 5.37 (2H, H-9, and H-10). The proton decoupled ^13^C-NMR (101 MHz) spectrum (Figure S2) and DEPT-135 (Figure S3) revealed the presence of 18 well-resolved carbon signals including methyl carbon at *δ* 14.3 (C-18), 14 methylene carbons at *δ* 22.9 (C-17), *δ* 24.8 (C-3), *δ* 27.4 (C-8 and C-11), *δ* 29.2–32.1 (C-4-C-7 and C-12-C-17), *δ* 34.1 (C-2), two methine carbons at *δ* 130.1 (C-9 and C-10), and a quaternary carbon corresponds to carbonyl carbon at *δ* 179.8 (C-1). The NMR data of compound **1** are consistent with the NMR data reported for oleic acid [24].

Compound **2** was obtained as a white solid with a melting point of 62–64°C. TLC gave a spot at an *R*_*f*_ value of 0.3 using *n*-hexane: EtOAc (4 : 1) as mobile phase. The ^1^H-NMR (400 MHz, CDCl_3_) spectrum (Figure S4) showed the presence of 31 protons, including a methyl proton at *δ* 0.88 (3H, *t*, *J* = 7.3 Hz, H-16), 11 methylene protons with broad singlet at *δ* 1.25 (22H, br. s, H-4-H-14), and three additional methylene protons at *δ* 1.30 (2H, m, H-15), *δ* 1.62 (2H, m, H-3), and *δ* 2.35 (2H, *t*, *J* = 7.3 Hz, H-2). The proton decoupled ^13^C-NMR (101 MHz) spectrum (Figure S5) with the aid of DEPT-135 (Figure S6) revealed the presence of 16 well-resolved carbon signals including a methyl at *δ* 14.3 (C-18), 14 methylenes at *δ* 22.9 (C-15), *δ* 24.8 (C-3), *δ* 29.2–29.9 (C-4-C-13), *δ* 32.1 (C-14), and *δ* 34.2 (C-2), and a quaternary carbon corresponds to carbonyl carbon at *δ* 180.0 (C-1). The NMR data of compound **2** are consistent with the NMR data reported for palmitic acid [25].

Compound **3** was obtained as a white crystal. TLC showed a spot at an *R*_*f*_ value of 0.4 using *n*-hexane: EtOAc (4 : 1) as the mobile phase. The ^1^H-NMR spectrum (Figure S7) showed a mixture of compound **3** (major, 61.3%) and compound **4** (minor, 38.7%). The percentage was calculated using the area of the proton representing both compounds (H-6) and representing only stigmasterol (H-22 or H-23) (Figure S7). In general, 96 protons and 58 carbons were identified, including 12 methyl carbons, 20 methylene carbons, 20 methine carbons, and six quaternary carbons. The NMR data generated for compounds **3** and **4** (Figures S7–S9) were compared with the reported data for stigmasterol and *β*-sitosterol and found in good agreement [26]. The complete assignment of all NMR data is presented in Table 1.

Compound **5** was observed as yellowish jelly and showed a TLC spot at *R*_*f*_ 0.7 using *n*-hexane: EtOAc: CHCl_3_: MeOH (5 : 3 : 1 : 1) as the mobile phase. 40 signals were identified for each proton and carbon from NMR spectral data (Figures S10–S12). The ^1^H-NMR (Figure S10) spectrum data indicated that an ABX set of signals at *δ* 2.00–2.05 (2H, m, H-8‴), 2.70 (2H, *t*, *J* = 7.8 Hz, H-7″), and 4.23 (2H, *t*, *J* = 6.5 Hz, H-9″) was assigned for methylene carbons at *δ* 30.9, 32.3, and 63.9, respectively. The observation of an ABC set of signals is further supported by diastereotopic protons (H-9′) at *δ* 4.41 (1H, dd, *J* = 8, 12 Hz) and 4.58 (1H, dd, *J* = 4, 12 Hz) assigned for the carbon signal at 65.6, as well as methine protons that correspond to carbon signal at *δ* 50.9 and 89.1, respectively, appeared at 3.84–3.92 (1H, m, H-8′) and 5.50 (1H, d, *J* = 7.8 Hz, H-7′). Two trans-coupled protons are observed at *δ* 6.23 (1H, d, *J* = 15.9 Hz, H-8) and 7.47 (1H, d, *J* = 15.9 Hz, H-7) and also at 6.29 (1H, d, *J* = 15.9 Hz, H-8‴) and 7.59 (1H, d, *J* = 15.9 Hz, H-7‴), which were assigned to carbon signals at *δ* 114.9, 145.6, 115.6, and 145.0, respectively. The presence of a tetrasubstituted benzene ring is indicated by the two broad singlet protons identified at *δ* 6.69 (1H, H-2″) and 6.71 (1H, H-6′), which are meta-aromatic protons linked to the carbon signal at *δ* 112.7 and 116.3, respectively. At *δ* 5.64 (1H, 4′-OH) and 5.91 (2H, 4, 4″-OH), two additional broad singlet protons were observed as a result of the three hydroxyl protons involved in hydrogen bonding. The methoxy proton signals from *δ* 3.84–3.92 were assigned to carbon signals at *δ* 55.9–60.0.  Table S-1 provides a summary of all the NMR spectral data for compound **5**. Similar NMR spectral data have been documented for a compound called boehmenan isolated from *Hibiscus cannabinus* and *Ochroma lagopus* Swartz [27, 28].

Compound **6** was obtained as a yellowish jelly showing the TLC spot at *R*_*f*_ value 0.5 with *n*-hexane: EtOAc: CHCl_3_: MeOH (5 : 3 : 1 : 1) as a mobile phase. The NMR spectral data revealed that the sample is a mixture of compound **6** (major) and compound **7** (minor) (Figures S13–S15). The complete assignment of the NMR spectral data is given in Tables 2 and 3. The two compounds are almost similar in their spectral data except signal due to H-7′ which was observed at *δ* 4.92 and 4.94 ppm assigned to compound **6** and compound **7**, respectively. Literature reports showed that the stereochemistry of these compounds at H-7 is different [28]. In this regard, H-7′ appeared as a singlet at *δ* 4.92 (equatorial with H-8′) in compound **6**; however, it appears as a doublet (*J* = 8.1 Hz) at *δ* 4.94 (axial with H-8′) in compound **7**.

However, in the current report, due to the complexity of the ^1^H-NMR spectrum, it was difficult to identify the multiplicity of H-7′ for compounds **6** and **7**. Using the chemical shift difference of H-7′ in compounds **6** and **7** (Figure S13), the area under integration of H-7′ was determined. The result indicated that the compositions of compounds **6** and **7** in the mixture were calculated as 57% and 43%, respectively. From *Ochroma lagopus* Swartz and *Euphorbia sikkimensis* plants, similar NMR spectral data of compounds **6** and **7** were reported for the compounds erythro-carolignan E and threo-carolignan E, respectively [27, 29]. Hence, the NMR spectral data of compounds **6** and **7** were compared (Tables 2 and 3) with the same compounds reported in the literature and found in good agreement [28].

### 3.2. Antibacterial Activities

#### 3.2.1. Disk Diffusion Method

The results of an *in vitro* antibacterial assay performed using a disk diffusion method against clinical isolate bacteria including *S*. *aureus*, *S*. *pyogenes*, *E*. *coli*, and *P*. *aeruginosa* are shown in Table 4. As indicated, at 100 mg/mL, the MeOH extract showed a better inhibition zone with a diameter of 12.30 ± 0.21 mm against *S*. *pyogenes*. It showed the lowest inhibition zone with a diameter of 8.20 ± 0.12 mm against *S*. *aureus*. On the contrary, the extract of *n*-hexane: EtOAc (1 : 1) demonstrated the maximum inhibition zone with a diameter of 15.20 ± 0.17 mm against *P*. *aeruginosa* and showed the least inhibition zone (8.40 ± 0.26) against *E*. *coli*.

In order to find out the active constituents, the isolated compounds were also assessed for their antibacterial activity. Among the isolated compounds, compound **3** had an inhibition zone of 11.10 ± 0.17, 10.50 ± 0.21, 10.30 ± 0.24, and 10.60 ± 0.55 against *S*. *aureus, S*. *pyogenes, E*. *coli,* and *P*. *aeruginosa*, respectively. The result turned out to be close to the activity displayed by ciprofloxacin. Compound **6** showed a comparatively better inhibition zone with a diameter of 10.40 ± 029 mm against *E*. *coli*. The activity displayed by the root extract of *C*. *hirsuta* in the present study was comparable with antibacterial activity reported in the literature carried out on the CHCl_3_ and MeOH extracts of *C*. *hirsuta leaves* where the CHCl_3_ extract showed an inhibition zone of 12.33, 10.70, and 10.26 mm and the MeOH extract showed an inhibition zone of 8.50, 9.30, and 8.00 mm against *P*. *aeruginosa*, *E*. *coli*, and *S*. *aureus*, respectively [11]. This confirms that the *n*-hexane: EtOAc (1 : 1) extract of the plant contains bioactive substances that have better activities against *P*. *aeruginosa* than against the other test bacteria.

#### 3.2.2. Minimum Inhibitory Concentration (MIC) of Extracts

The results of the MIC assay of the plant root extracts performed using the agar dilution method against two Gram-positive clinical isolates (*S*. *aureus* and *S*. *pyogenes*) and two Gram-negative clinical isolates (*E*. *coli* and *P*. *aeruginosa*) are shown in Table 5. As indicated, the MIC results were identified to be greater than or equal to 50 mg/mL for both MeOH and *n*-hexane: EtOAc (1 : 1) extracts against the four bacterial strains. Unlike the MIC of the standard drug, ciprofloxacin was determined to be less than 15.625 *μ*g/mL against all selected bacteria. Comparable results were observed from the MIC evaluation conducted on the MeOH and CHCl_3_ extracts of the plant leaves against *S*. *aureus*, *E*. *coli*, and *P*. *aeruginosa* wherein the maximum MIC value was found to be 60 mg/mL and the minimum MIC value was 30 mg/mL [12].

### 3.3. Radical Scavenging Activities

The result of the ability to inhibit the DPPH radical of extract and isolated compounds is presented in Table 6. At 62.5 *μ*g/mL, the percent of DPPH radical inhibited using extracts of methanol and *n*-hexane: EtOAc (1 : 1) was 56.7% and 41.7%, respectively. Compounds **3**, **5**, and **6** inhibited 30.3, 92.1, and 86.5% of the DPPH radical, respectively. Compound **5** showed potent DPPH radical scavenging activity with an IC_50_ value of 2.1 *μ*g/mL which is comparable with ascorbic acid with an IC_50_ value of 0.5 *μ*g/mL. The IC_50_ values of compounds **3** and **6** were 19.4 *μ*g/mL and 7.4 *μ*g/mL, respectively. Compounds **5** and **6** showed higher radical scavenging activities than the extracts and other isolated compounds at 62.5 *μ*g/mL, compared to the radical scavenging ability of ascorbic acid. The strong DPPH radical scavenging activities of compounds **5** and **6** could be related to their number of hydroxyl groups which implies that phenolic compounds exhibit potent DPPH radical scavenging activities [30]. It was found that the radical scavenging activity displayed by MeOH extract of the root of the plant was superior compared with literature reported for the same plant [2].

### 3.4. *In Silico* Molecular Docking Analysis of the Isolated Compounds

The molecular docking study of isolated compounds was carried out against targets, *E*. *coli* DNA gyrase B (PDB ID: 7P2M) and the *Pseudomonas* quinolone signal A, PqsA (PDB ID: 5OE4), to evaluate *in silico* antibacterial activities and against Pyruvate kinase M2, PKM2 (PDB ID: 4G1N), and human topoisomerase *IIβ* (PDB ID: 3QX3) to study the *in silico* anticancer activities of isolated compounds.

The results of docking analysis against *E*. *coli* DNA gyrase B (PDB ID: 7P2M) and *Pseudomonas* quinolone signal A, PqsA (PDB ID: 5OE4) were compared with the standard antimicrobial, ciprofloxacin (Table 7).

The docking study with 7P2M revealed that the binding affinity of the isolated compounds ranged from −7.9 to −6.4 kcal/mol compared to the binding affinity of ciprofloxacin (−7.4 kcal/mol). The highest docking score was exhibited by compound **7** (−7.9 kcal/mol) while the lowest was exhibited by compound **5** (−6.4 kcal/mol). As indicated, except compound **4**, compounds **3, 5, 6,** and **7** displayed hydrogen bonding with 7P2M through different amino acid residues. Compound **3** interacted through one hydrogen bonding, and each of the compounds **5**–**7** interacted using three hydrogen bonds with 7P2M. Based on the binding affinities calculated, compared to ciprofloxacin, compounds **3**, **6**, and **7** can be considered as good antibacterial activity against *E*. *coli*.

**Figure 2:**
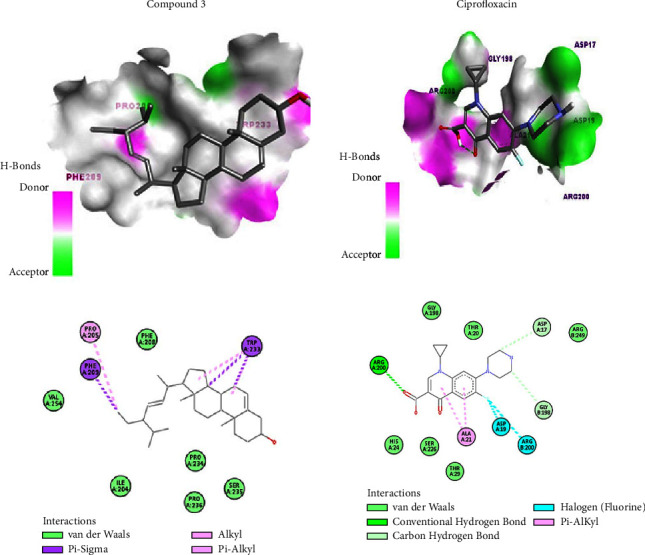
The 3D (top) and 2D (bottom) binding interactions of selected (compound **3**) and ciprofloxacin against the *Pseudomonas* quinolone signal A, PqsA (PDB ID: 5OE4).

The docking analysis against 5OE4 indicated that the binding affinity of isolated compounds ranged from −9.0 to −8.3 kcal/mol compared to the docking score of ciprofloxacin (−7.2 kcal/mol). The highest binding affinity was exhibited by compound **3** (−9.0 kcal/mol), while the lowest binding affinity was exhibited by compound **5** (−8.3 kcal/mol). Compound **3** showed the highest docking score (−9.0 kcal/mol) without hydrogen bonding (Figure 2), while compound **5** showed hydrogen bonding through Ser-226, Gly-198, and Arg-200 amino acid residues which interacted with the lowest docking score (−6.4 kcal/mol). All the tested compounds showed greater affinity for PqsA compared to that of ciprofloxacin. In general, the *in silico* antibacterial study indicated that compound **3** comparatively showed good antibacterial activity compared to the rest of the isolated compounds. Therefore, the 2D and 3D interaction of compound **3** with PqsA is selectively compared to the 2D and 3D interaction of ciprofloxacin (Figure 2). Unlike compound **3**, ciprofloxacin interacted with PqsA using hydrogen bonding through Arg-200 amino acid residues in addition to hydrophobic, electrostatic, and van der Waals interaction. The 2D and 3D interaction of compounds **3–7** with 7P2M and 5OE4 is shown in Figures S16–S17.

The results of the *in silico* antibacterial studies support the *in vitro* antibacterial activities, and therefore, the isolated compounds can be considered as potential antibacterial agents. Furthermore, it is noted that ciprofloxacin targets both DNA gyrase (topoisomerase II) and topoisomerase IV [31]. Therefore, the inconsistency of the result for ciprofloxacin observed in *in vitro* with the *in silico* antibacterial activity test might be related to the mechanism of action of ciprofloxacin, as the *in silico* antibacterial activity evaluation was performed against a specific enzyme. Thus, it can be concluded that the correlation between the number of hydrogen bonds and the values of the docking score is not proportional. As observed in this report, a docking result containing more hydrogen bonding can be found with a low binding affinity. For example, the results reported for molecular docking studies conducted against *Plasmodium falciparum* hexose transporter 1 (PfHT1) protein demonstrated that the ligand-protein complex with the highest number of hydrogen bonding was found with the lowest docking score [32].

The result of docking analysis against pyruvate kinase M2, PKM2 (PDB ID: 4G1N), and human topoisomerase *IIβ* (PDB ID: 3QX3) was compared with the result of the standard anticancer drug, abiraterone (Table 8).

**Figure 3:**
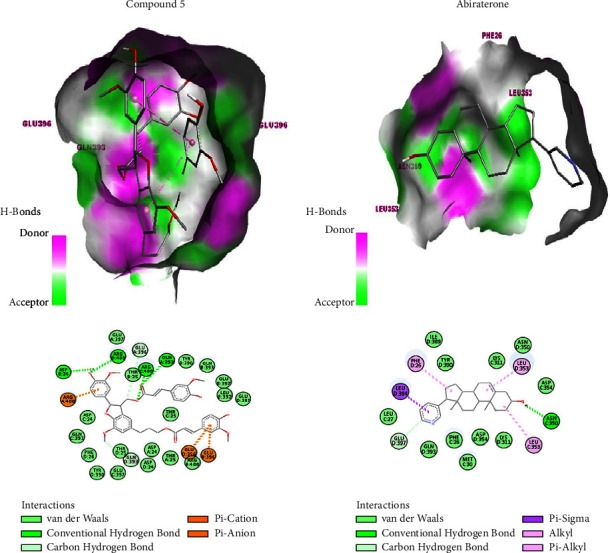
The 3D (top) and 2D (bottom) binding interactions of compound **5** and abiraterone against pyruvate kinase M2, PKM2 (PDB ID: 4G1N).

The docking with 4G1N showed that the binding affinity of isolated compounds ranged from −8.5 to −7.1 kcal/mol compared to the docking score of abiraterone (−9.5 kcal/mol). The highest binding affinity (−8.5 kcal/mol) was shown by compound **5** while the lowest (−7.1 kcal/mol) was shown by compound **3**. The hydrogen bond interaction was not observed between compound **3** (−7.1 kcal/mol) and 4G1N. Compound **3** interacted only using hydrophobic, electrostatic, and van der Waals interactions. Compounds **4**, **5, 6,** and **7** showed hydrogen bonding interaction in addition to hydrophobic, electrostatic, and van der Waals interaction. Compound **4** (−7.5 kcal/mol) demonstrated hydrogen bonding through the amino acid residue tyr-390, while compound **5** (−8.5 kcal/mol) demonstrated hydrogen bonding through the amino acid residues Asp-24, Arg-400, Gln-393, and Arg-400 (Figure 3). Similarly, compound **6** (−8.3 kcal/mol) demonstrated hydrogen bonding via the amino acid residues Arg-400 and Glu-396, while compound **7** (−8.1 kcal/mol) demonstrated hydrogen bonding with 4G1N through the amino acid residues Arg-400, Arg-399, Arg-447, and His-391. As indicated, both compounds **5** and **7** showed the same number of hydrogen bonds with different binding affinity. Furthermore, compound **6** showed a smaller number of hydrogen bonding interactions than compound **7**, but it showed a higher affinity against 4G1N. Among all isolated compounds, compared to the standard anticancer drug abiraterone, compound **5** was predicted to have better anticancer activities against 4G1N.

The docking study with 3QX3 showed that the binding affinity of isolated compounds ranged from −8.5 to −7.9 kcal/mol compared to the binding affinity of abiraterone (−9.3 kcal/mol). The highest docking score (−8.5 kcal/mol) was seen with compound **5** while the lowest (−7.9 kcal/mol) was shown with compound **3**. Compound **3** interacted with 3QX3 through only hydrophobic, electrostatic, and van der Waals interaction. However, in addition to the hydrophobic, electrostatic, and van der Waals interaction, compounds **4**, **5**, **6**, and **7** displayed a hydrogen bonding interaction with 3QX3. Compound **4** showed one hydrogen bonding through His-775, while compound **5** showed a hydrogen bonding through Arg-688, Arg-743, His-775, and Lys-744. Similarly, compound **6** showed hydrogen bonding through amino acid residues Arg-743, Phe-738, and Arg-688, while compound **7** showed hydrogen bonding through amino acid residues Phe-738, Arg-743, Arg-688, Glu-728, Lys-744, and Ser-725. As indicated, compound **5**, with a higher binding affinity (−8.5 kcal/mol), showed four hydrogen bonding interactions while compound **7**, with a lower binding affinity (−8.2 kcal/mol), showed six hydrogen bonding interactions. Of all isolated compounds, compared to the activities of the standard anticancer drug, abiraterone, compound **5** was predicted to show better anticancer activity against 3QX3.

In conclusion, from *in silico* anticancer activity studies, compound **5** showed good activities against both cancer-causing enzymes, 4G1N and 3QX3, and hence, selectively the 2D and 3D interaction of compound **5** with 4G1N is compared with the 2D and 3D interaction of abiraterone (Figure 3). For the rest of the isolated compounds, the 2D and 3D interactions with 4G1N and 3QX3 are shown in Figures S18 and S19, respectively.

### 3.5. *In Silico* Pharmacokinetics and Toxicity of the Isolated Compounds

Table 9 shows the results of the drug-like properties calculated from the isolated compounds using SwissADME according to Lipinski's rule. As indicated, compounds **3** and **4** violate Lipinski's rule similar to the standard anticancer drug, abiraterone. However, compounds **5** and **6** showed two violations of Lipinski's rule implying that they cannot be administered orally as a drug [22]. From Pro-Tox II analysis, acute oral toxicity, organ toxicity, and toxicological endpoints were predicted. Compounds **3**, 4, **5**, and **6** showed predicted class 4 toxicity and have shown immunotoxicity. In the same way, the acute oral toxicity prediction result for standard antibiotics, ciprofloxacin, indicated predicted toxicity class 4 and has shown only mutagenicity. Also, the standard anticancer drug, abiraterone, has shown a class 4 prediction of toxicity with the property of immunotoxicity. Therefore, none of the isolated compounds was shown to have acute toxicity due to the predicted LD_50_ which is greater than five [23].

## 4. Conclusion

In this study, seven compounds were identified from the roots of *C*. *hirsuta*. Compounds **5**–**7** were reported for the first time from the plant and the genus of the plant. For the secondary metabolites, the *in vitro* antibacterial activities conducted using the disk diffusion method against four bacteria were validated by using *in silico* molecular docking analysis. Compound **3** exhibited relatively good antibacterial activity against *S. aureus* and *P*. *aeruginosa* bacteria, while compound **6** showed better activity against *E*. *coli*. The MIC value of the extracts was greater than or equal to 50 *μ*g/mL against all bacteria. The extract of *n*-hexane: EtOAc (1 : 1) from the root of *C*. *hirsuta* contains phytochemical components which are the most potent antibacterial agents against Gram-negative bacteria, *P*. *aeruginosa*. Therefore, a high yield of the *n*-hexane: EtOAc (1 : 1) extract must be further studied to isolate a potent antibacterial agent against Gram-negative bacteria. On the contrary, even though two of Lipinski's rules of five were violated, compounds **5** and **6** were considered strong DPPH radical scavengers, and therefore, compounds **5** and **6** should be modified according to Lipinski's rules of five so that they could be used as lead compounds in the development of anticancer drugs. Furthermore, phytochemicals isolated from the root of the plant and which showed *in vitro* antibacterial activities and *in silico* anticancer activities are substantial evidence for the traditional uses of the plant to treat diseases caused by bacteria and cancer-related diseases. In addition, we recommend toxicity and *in vivo* assays for compounds **5**–**7** that were isolated from the root of *C*. *hirsute* to evaluate their potential as antibacterial and anticancer agents.

## Figures and Tables

**Table 1:** ^1^H-NMR (400 MHz, CDCl_3_) and ^13^C-NMR (101 MHz) of compounds **3** and **4** and reported NMR data for stigmasterol and *β*-sitosterol.

C no	Compound **3**		Stigmasterol [26]	Compound **4**		*β*-sitosterol [26]
	*δ* ^1^H	*δ* ^13^C	*δ* ^13^C	*δ* ^1^H	*δ* ^13^C	*δ* ^13^C
1	1.85 & 1.08 (br. s, 2H)	37.4	37.2	1.85 &1.08 (br. s, 2H)	37.4	37.2
2	1.83 (br. s, 2H)	31.8	31.6	1.83 &1.52 (br. s, 2H)	31.8	31.9
3	3.53 (m, 1H)	72.0	71.9	3.53 (br. s, 1H)	72.0	71.8
4	2.27 & 2.24 (br. s, 2H)	42.4	42.2	2.23 &2.27 (br. s, 2H)	42.5	42.3
5	—	140.9	140.7	—	140.9	140.7
6	5.36 (br. s, 1H)	121.9	121.7	5.36 (br. s, 1H)	121.9	121.7
7	2.00 &1.50 (br. s, 2H)	32.1	31.9	2.00 (br. s, 2H)	32.1	31.9
8	1.50 (1H)	32.1	31.9	1.50 (br. s, 1H)	32.1	32.4
9	0.93 (br. s, 1H)	50.3	50.1	0.93 (br. s, 1H)	50.3	50.1
10	—	36.7	36.5	—	36.7	36.5
11	1.50 (br. s, 2H)	21.2	21.0	1.50 (br. s, 2H)	21.2	21.1
12	2.00 &1.16 (br. s, 2H)	39.9	39.7	1.16 & 1.19 (br. s, 2H)	39.9	39.8
13	—	42.4	42.4	—	42.5	42.3
14	1.01 (br. s, 1H)	57.0	56.8	1.06 (br. s, 1H)	57.0	56.9
15	1.57 & 1.16 (br. s, 2H)	24.5	24.4	1.43 & 1.16 (br. s, 2H)	24.5	25.5
16	1.63 & 1.25 (br. s, 2H)	29.2	28.9	1.85 & 1.25 (br. s, 2H)	28.4	28.2
17	1.16 (br. s, 1H)	56.1	56.0	1.11 (br. s, 1H)	56.1	56.1
18	0.82 (s, 3H)	12.1	11.8	0.68 (s, 3H)	12.2	12.2
19	1.01 (br. s, 3H)	19.2	19.1	1.01 (br. s, 3H)	19.5	19.4
20	2.04 (1H)	40.6	40.5	1.38 (br. s, 1H)	36.3	35.9
21	1.03 (3H)	21.4	21.5	0.92 (br. s, 3H)	19.2	18.6
22	5.14 (m, 1H)	138.5	138.3	1.34 & 1.01 (br. s, 2H)	34.1	33.9
23	5.02 (m, 1H)	129.4	129.3	1.18 & 1.16 (br. s, 2H)	26.2	26.1
24	1.53 (1H)	51.4	51.2	0.93 (br. s, 1H)	46.0	45.8
25	1.45 (1H)	36.3	36.1	1.67 (br. s, 1H)	29.3	29.1
26	0.84 (br. s, 3H)	20.0	19.8	0.86 (br. s, 3H)	21.2	21.0
27	0.70 (br. s, 3H)	19.0	18.9	0.86 (br. s, 3H)	19.0	18.6
28	1.43 &1.16 (br. s, 2H)	25.6	25.4	1.28 (br. s, 2H)	23.2	22.9
29	0.68 (br. s, 3H)	12.4	12.0	0.86 (br. s, 3H)	12.0	12.0

**Table 2:** ^1^H-NMR (400 MHz, CDCl_3_) and ^13^C-NMR (101 MHz) of compound **6** and NMR data reported for erythro-carolignan E.

C no	Compound **6**		Erythro-carolignan E [28]
	*δ* ^1^H (*J* in Hz)	*δ* ^13^C	*δ* ^13^C
1	—	127.1	126.9
2	6.96–7.04 (m, 1H)	109.5	109.3
3	—	146.9	146.7
4	—	148.2	148.0
5	6.92 (br. s, 1H)	114.9	114.7
6	6.96–7.04 (m, 1H)	123.3	123.2
7	7.51 (d, *J* = 15.9, 1H)	145.4	145.2
8	6.25 (d, *J* = 15.9, 1H)	115.1	114.9
9	—	167.3	167.1
1′	—	131.3	131.0
2′	6.96–7.04 (m, 1H)	109.0	108.8
3′	—	146.8	146.6
4′	—	145.3	145.0
5′	6.88 (d, *J* = 8.1, 1H)	114.3	114.1
6′	6.84 (br. s, 1H)	119.4	119.2
7′	4.92 (br. s, 1H)	72.2	72.0
8′	4.45–4.52 (m, 1H)	84.7	84.5
9′	4.22–4.32 (m, 1H) 44.45–4.50 (m, 1H)	62.8	62.6
1″	—	137.6	137.4
2″	6.72–6.79 (m, 1H)	112.5	112.3
3″	—	151.5	151.3
4″	—	145.3	145.0
5″	6.84 (d, *J* = 8.2, 1H)	120.9	120.7
6″	6.72–6.79 (m, 1H)	121.3	121.1
7″	2.70 (t, *J* = 7.8, 2H)	32.2	32.0
8″	2.00–2.04 (br. s, 2H)	30.5	30.3
9″	4.23 (t, *J* = 6.2, 2H)	63.8	63.6
1‴	—	127.0	126.8
2‴	6.96–7.04 (m, 1H)	109.5	109.3
3‴	—	146.9	146.7
4‴	—	148.2	148.0
5‴	6.91 (m, 1H, m)	114.9	114.7
6‴	7.08 (br. s, 1H)	123.2	123.0
7‴	7.62 (d, *J* = 15.9, 1H)	145.1	144.9
8‴	6.32 (d, *J* = 15.9, 1H)	115.5	115.3
9‴	—	167.5	167.3
3-OCH_3_	3.92 (br. s, 3H)	56.1	55.9
3′-OCH_3_	3.88 (br. s, 3H)	56.1	55.9
3″-OCH_3_	3.88 (br. s, 3H)	56.0	55.8
3‴OCH_3_	3.93 (br. s, 3H)	56.1	55.9
4-OH	5.61 (br. s, 1H)	—	—
4′-OH	5.59 (br. s, 1H)	—	—
4″-OH	5.61 (br. s, 1H)	—	—

**Table 3:** ^1^H-NMR (400 MHz, CDCl_3_) and ^13^C-NMR (101 MHz) of compound **7** and NMR data reported for threo-carolignan E.

C no.	Compound **7**		Threo-carolignan E [28]
	*δ* ^1^H (*J* in Hz)	*δ* ^13^C	*δ* ^13^C
1	—	127.1	126.9
2	7.02–7.07 (m, 1H)	109.5	109.3
3	—	147.0	146.8
4	—	148.2	148.0
5	6.79–6.95 (m, 1H)	114.9	114.7
6	7.02–7.07 (m, 1H)	123.3	123.2
7	7.51 (d, *J* = 15.9, 1H)	145.6	145.5
8	6.28 (d, *J* = 15.9, 1H)	114.9	114.7
9	—	167.0	166.8
1′	—	131.3	131.1
2′	6.79–6.95 (m, 1H)	109.4	109.2
3′	—	146.9	146.7
4′	—	145.8	145.6
5′	6.79–6.95 (m, 1H)	114.5	114.3
6′	6.79–6.95 (m, 1H)	120.6	120.4
7′	4.94 (br. s, 1H)	74.6	74.4
8′	4.18–4.27 (m, 1H)	86.5	86.3
9′	4.09–4.17 (m, 1H) 4.35–4.38 (m, 1H)	63.2	63.0
1″	—	137.6	137.4
2″	6.77 (m, 1H)	112.5	112.3
3″	—	150.9	150.7
4″	—	146.2	146.0
5″	7.02–7.07 (m, 1H)	120.8	120.6
6″	6.72–6.79 (m, 1H)	121.2	121.0
7″	2.70 (t, *J* = 7.8, 2H)	32.2	32.0
8″	2.00–2.04 (br. s, 2H)	30.6	30.4
9″	4.23 (t, *J* = 6.2, 2H)	63.8	63.6
1‴	—	127.0	126.7
2‴	7.02–7.07 (m, 1H)	109.5	109.3
3‴	—	147.0	146.8
4‴	—	148.3	148.1
5‴	6.79–6.95 (m, 1H)	114.9	114.7
6‴	7.08 (br. s, 1H)	123.2	123.0
7‴	7.62 (d, *J* = 15.9, 1H)	145.3	145.0
8‴	6.32 (d, *J* = 15.9, 1H)	115.5	115.3
9‴	—	167.5	167.3
3-OCH_3_	3.85 (br. s, 3H)	56.1	55.9
3′-OCH_3_	3.93 (br. s, 3H)	56.1	55.9
3″-OCH_3_	3.90 (br. s, 3H)	56.0	55.8
3‴-OCH_3_	3.94 (br. s, 3H)	56.1	55.9
4-OH	5.89 (br. s, 1H)	—	—
4′-OH	5.61 (br. s, 1H)	—	—
4″-OH	5.89 (br. s, 1H)	—	—

**Table 4:** Bacterial growth inhibition zone of extracts and isolated compounds.

Test sample	Concentration (mg/mL)	Inhibition zone in mm (mean ± SD)			
		*S*. *aureus*	*S*. *pyogenes*	*E*. *coli*	*P*. *aeruginosa*
MeOH extract	100	8.20 ± 0.12	12.30 ± 0.21	9.20 ± 0.12	9.30 ± 0.22
	50	NI	8.20 ± 0.12	7.20 ± 0.12	NI

*n*-hexane: EtOAc (1 : 1) extract	100	11.20 ± 0.13	10.30 ± 0.25	8.40 ± 0.26	15.20 ± 0.17
	50	7.10 ± 0.13	8.30 ± 0.13	NI	10.20 ± 0.12

**3**	5	11.10 ± 0.17	10.50 ± 0.21	10.30 ± 0.24	10.60 ± 0.55
	2.5	7.27 ± 0.55	8.70 ± 0.22	8.20 ± 0.17	8.20 ± 0.17

**5**	5	9.07 ± 0.12	8.80 ± 0.17	9.10 ± 0.19	9.10 ± 0.12
	2.5	NI	NI	7.60 ± 0.13	NI

**6**	5	10.10 ± 0.10	10.20 ± 0.17	10.40 ± 0.29	10.20 ± 0.22
	2.5	8.27 ± 0.46	7.50 ± 0.22	8.30 ± 0.16	8.40 ± 0.24

Ciprofloxacin	0.5	18.42 ± 0.26	19.93 ± 0.21	20.00 ± 0.21	22.18 ± 0.22

*Note.* NI: no inhibition.

**Table 5:** MIC of MeOH and *n*-hexane: EtOAc (1 : 1) extracts of *C*. *hirsuta* root.

S/N	Bacteria	MIC of extracts (mg/mL)		Ciprofloxacin (mg/mL)
		MeOH extract	*n*-hexane: EtOAc (1 : 1) extract	
1	*S*. *aureus*	>50	>50	≤0.015625
2	*S*. *pyogenes*	50	50	
3	*E*. *coli*	50	50	
4	*P*. *aeruginosa*	>50	50	

**Table 6:** DPPH radical scavenging activity of the extracts and isolated compounds.

Sample	% inhibition of DPPH radical					IC_50_ value (*μ*g/mL)
	1000	500	250	125	62.5	
Ascorbic acid	97.9 ± 0.06	97.3 ± 0.05	97.2 ± 0.05	97.0 ± 0.1	96.9 ± 0.06	0.5
MeOH extract	91.1 ± 0.06	82.8 ± 0.2	73.2 ± 0.06	60.7 ± 0.06	56.7 ± 0.06	49.4
*n*-hexane: EtOAc (1 : 1) extract	81.5 ± 0.12	78.5 ± 0.15	67.2 ± 0.1	54.6 ± 0.12	41.7 ± 0.2	73.1
**3**	40.3 ± 0.2	35.2 ± 0.2	33.1 ± 0.2	31.9 ± 0.2	30.3 ± 0.3	19.4
**5**	95.4 ± 0.1	94.5 ± 0.2	94.0 ± 0.1	93.3 ± 0.1	92.1 ± 0.1	2.1
**6**	95 ± 0.2	94.3 ± 0.2	94.2 ± 0.2	88.0 ± 0.1	86.5 ± 0.2	7.4

*Note.* The %inhibition was reported as mean ± SD.

**Table 7:** Molecular docking results of isolated compounds against *E*. *coli* DNA gyrase B (PDB ID: 7P2M) and *Pseudomonas* quinolone signal A, PqsA (PDB ID: 5OE4) (binding affinity in kcal/mol).

Target	Ligand	Binding affinity	H-bonding	Hydrophobic and electrostatic	Van der Waals
7P2M	**3**	−7.8	Thr-165	Alkyl: Ile-78, 94	Leu-98, Val-93, 97, 120, Gly-119, 75, 77, Asn-46, Asp-73, Glu-50, Arg-76, Pro-79
	**4**	−6.8	—	Pi-sigma: Phe-196; alkyl and pi-alkyl: Arg-192, Leu-197, Phe-196	Glu-193, Ser-195, His-217, Phe-216, Tyr-218
	**5**	−6.4	Asn-46, Arg-76, Asp-49	Pi-sigma: Ile-78; alkyl and pi-alkyl: Ala-47, Ile-94, Val-120, Ala-53; carbon hydrogen bond: Asp-73	Gly-119, Val-93, 167, 97, Pro-79, Thr-165, Met-95, Glu-50, Leu-98
	**6**	−7.6	Arg-76, Asn-46, Val-120	Pi-sigma: Thr-165; alkyl and pi-alkyl: Val-167, 120, 43, Ile-78, 84; carbon hydrogen bond: Val-71, 97, Thr-165	Pro-79, Arg-136, Ala-90, 53, 47, Gly-77, 119, Asp-49, 73, Val-93, Leu-98, Met-166, Glu-50
	**7**	−7.9	Asp-73, Val-120, Ser-121	Pi-sigma: Thr-165; alkyl and pi-alkyl: Ala-47, Val-43, 97, 93, Ile-94, 78; carbon hydrogen bond: Val-71, Thr-165, Asp-73, Gly-119	Val-167, Asn-46, Asp-49, His-83, Arg-76, 136, Pro-79, Gly-77, Glu-50
	Ciprofloxacin	−7.4	Arg-76	Halogen: Asp-73; amide-pi stacked: Gly-77; alkyl and pi-alkyl: Ile-78, 94, Pro-79; carbon hydrogen bond: Asp-73	Arg-136, Ala-47, Asn-46, Val-43, 167, 120, Glu-50, Trh-165

5OE4	**3**	−9.0	—	Pi-sigma: Phe-209, Trp-233; alkyl and pi-alkyl: Pro-205, Trp-233	Val-254, Ile-204, Pro-234, 236, Ser-235, Phe-208
	**4**	−8.8	—	Pi-sigma: Phe-209, Trp-233; alkyl and pi-alkyl: Pro-205, Phe-209, Trp-233	Val-254, Pro-234, Phe-208, Lys-206, Ile-204
	**5**	−8.3	Ser-226, Gly-198, Arg-200, 200	Pi-anion: Asp-17, 19; pi-sigma: Ala-21; alkyl and pi-alkyl: Arg-249, 200, Ala-21; pi-donor and carbon hydrogen bond: Asp-199, 19, Ala-21, Arg-247	Thr-29, 29, 20, 20, His-24, 24, Asp-199, 19, 17, Gly-198, Pro-248, Ser-31, 226
	**6**	−8.7	Arg-200, 200, His-24, Ser-226	Pi-anion and cation: Arg-200, Asp-19; alkyl, pi-alkyl, and pi-sigma: Arg-249, Ala-21; carbon and pi-donor hydrogen bond: Gly-198, 224, Arg-247, Asp-19, Ala-21	Pro-248, Thr-20, 20, 29, 29, Gly-198, Ala-125, 21, 197, Asp-199, 19, 17, His-24
	**7**	−8.4	Gln-34, Ser-31, Arg-200, 200, Ala-21	Pi-cation: Arg-200; pi-sigma: Thr-29; alkyl and pi-alkyl: Ala-245, 21, 21; carbon hydrogen bond: His-24, Asp-117	Leu-228, 30, Phe-246, His-24, Thr-29, 20, 20, Ser-31, Asp-19, 19, Gly-198, 27
	Ciprofloxacin	−7.2	Arg-200	Halogen: Asp-19, Arg-200; alkyl: Ala-21; carbon hydrogen bond: Gly-198, Asp-17	His-24, Ser-226, Thr-29, 20, Arg-249, Gly-198

**Table 8:** Molecular docking results of isolated compounds against pyruvate kinase M2, PKM2 (PDB ID: 4G1N), and human topoisomerase *IIβ* (PDB ID: 3QX3) (binding affinity in kcal/mol).

Target	Ligand	Binding affinity	H-bonding	Hydrophobic and electrostatic	Van der Waals
4G1N	**3**	−7.1	—	Alkyl: Pro-403, Met-22, Leu-392	Leu-401, Ala-402, 21, Ile-404, Lys-422, Phe-421, 395, Arg-399, Glu-418, 400
	**4**	−7.5	Tyr-390	Pi-alkyl: Tyr-390	Gln-393, 393, 393, Arg-400, 400, 400, Thr-25, 25, Asp-24, 24, Glu-397, 396, 396, 396, Leu-392
	**5**	−8.5	Asp-24, Arg-400, 400, Gln-393	Pi-anion and pi-cation: Arg-400, Glu-396, 396; carbon hydrogen bond: Glu-396, Gln-393	Thr-25, 390, Gln-393, Glu-397, Leu-392, Arg-400, Asp-24, Tyr-390, Phe-26
	**6**	−8.3	Arg-400, 400, Glu-396	Carbon hydrogen bond: Gly-393, 393, Thr-25; pi-anion: Glu-397, 396	Thr-25, 25, 25, Glu-396, 396, 397, 397, 397, Phe-26, 26, 26, 26, Asp-24, 24, 24, 24, Tyr-390, 390, 390, Gln-393, 393 Leu-392, Arg-400, 400
	**7**	−8.1	Arg-400, 399, 447, His-391	Pi-anaion: Asp-24; pi-sigma: Ile-404; pi-sulfur: Met-22; pi-pi T-shaped: Phe-421; alkyl and pi-alkyl: Ala-21, Lys-422, Ile-404; carbon hydrogen bond: Met-22	Arg-32, Leu-401, 392, 18, Glu-418, Ala-402, Pro-403
	Abiraterone	−9.5	Asn-350	Carbon hydrogen bond: Glu-397; pi-sigma: Leu-394; alkyl and pi-alkyl: Phe-26, Leu-353, 353	Leu-27, Ile-389, Tyr-390, Lys-311, 311, Asn-350, Asp-354, 354, Met-30, Phe-26, Gln-393

3QX3	**3**	−7.9	—	Alkyl and pi-alkyl: His-774, 775, Pro-740	Glu-853, 855, 728, Leu-845, Arg-743, Trp-856, His-1021, Asp-1020, Ser-733, Gly-1023, Phe-1019, Lys-744, Tyr-773, Ala-772
	**4**	−8.4	His- 775	Pi-sigma: Phe-1019; alkyl and pi-alkyl: Phe-1019, Pro-740, Leu-845	Gly-1023, Ser-733, Trp-856, Arg-743, 729, Glu-855, 853, 728, Lys-744, His-744
	**5**	−8.5	Arg-688, 743, His-775, Lys-744	Carbon hydrogen bond: Gly-1023, Asp-847, Asp-1020, Ser-733, Glu-728; pi-pi stacked: Phe-1019; alkyl and pi-alkyl: Val-852, Pro-740, Arg-729	Phe-738, Lys-739, Trp-856, Glu-855, 853, Gln-560, Ser-725, His-774, Leu-845, Arg-692, Met-1022
	**6**	−8.1	Arg-743, 688, Phe-738	Pi-cation: Arg-688; pi-pi stacked: Phe-1019; carbon hydrogen bond: Glu-728, Asp-1020; alkyl and pi-alkyl: Trp-856, Leu-721, 845, Pro-740, Arg-692	Ser-733, 725, Lys-739, Pro-732, 854, Glu-855, 853, Gln-560, Asn-724, Phe-684, Gly-1023
	**7**	−8.2	Phe-738, Arg-743, 688, Glu-728, Lys-744, Ser-725	Pi-anion: Glu-728; alkyl and pi-alkyl: Pro-740, Leu-845, pi-pi stacked: Phe-1019; carbon hydrogen bond: Asp-1020, 847, Glu-855, 728	Trp-856, Tyr-846, Val-852, Ala-772, His-775, Gln-560, Arg-729, Ile-731, Gly-1023, Lys-739, Ser-733, Pro-732
	Abiraterone	−9.3	Arg-743, Ser-733	Alkyl and pi-alkyl: Arg-692, Phe-1019, Pro-740; pi-cation: Arg-688	Gly-1023, Glu-855, 728, Trp-856, Lys-739, Phe-738, Ile-731, Pro-732, Arg-689

**Table 9:** Drug-likeness predictions of isolated compounds computed by SwissADME.

Ligand	Formula	MWT (g/mol)	NRB	NHA	NHD	TPSA (Å^2^)	Log P	Lipinski's rule of five
**3**	C_29_H_48_O	412.7	5	1	1	20.2	7.0	1
**4**	C_29_H_50_O	414.7	6	1	1	20.2	6.73	1
**5**	C_40_H_40_O_12_	712.7	17	12	3	159.4	5.4	2
**6**	C_40_H_42_O_13_	730.8	20	13	4	179.4	5.3	2
Ciprofloxacin	C_17_H_18_FN_3_O_3_	331.3	3	5	2	74.6	−1.1	0
Abiraterone	C_24_H_31_NO	349.5	1	2	1	33.1	4.4	1 (MLogP > 4.15)

*Note.* MWT: molecular weight; NRB: number of rotatable bonds; NHA: number of hydrogen acceptors; NHD: number of hydrogen donors; TPSA: total polar surface area.

## Data Availability

The data used to support the findings of this study are included in the article and provided as supportive information.

## Supplementary Materials

Supplementary Materials Figure S1: ^1^H-NMR spectra of compound **1**. Figure S2: ^13^C-NMR spectra of compound **1**. Figure S3: ^13^C DEPT-135 NMR spectrum of compound **1**. Figure S4: ^1^H-NMR spectra of compound **2**. Figure S5: ^13^C-NMR spectra of compound **2**. Figure S6: ^13^C DEPT-135 NMR spectrum of compound **2**. Figure S7: ^1^H-NMR spectra of compounds **3** and **4** and the enlarged region used to calculate the relative amount of compounds **3** and **4**. Figure S8: ^13^C-NMR spectra of compounds **3** and **4**. Figure S9: ^13^C DEPT-135 NMR spectrum of compounds **3** and **4**. Table S-1. ^1^H-NMR (400 MHz, CDCl_3_) and ^13^C-NMR (101 MHz) of compound **5** and NMR data reported for boehmenan. Figure S10: ^1^H-NMR spectra of compound **5**. Figure S11: ^13^C-NMR spectra of compound **5**. Figure S12: ^13^C DEPT-135 NMR spectrum of compound **5**. Figure S13: ^1^H-NMR spectra of compounds **6** and **7** and enlarged region used to calculate the relative amount of compounds **6** and **7**. Figure S14: ^13^C-NMR spectra of compounds **6** and **7**. Figure S15: ^13^C DEPT-135 NMR spectrum of compounds **6** and **7**. Figure S16 to 19: 2D and 3D interactions of isolated compounds with selected protein targets.
