# Supplementary material for: In Vitro Antibacterial, DPPH Radical Scavenging Activities, and In Silico Molecular Modeling of Isolated Compounds from the Roots of Clematis hirsuta
Source: Adv Pharmacol Pharm Sci. 2024 Mar 25;2024:3152929. doi: 10.1155/2024/3152929 (PMC10984721; doi:10.1155/2024/3152929)
Supplement: Supplementary Materials — Figure S1: 1H-NMR spectra of compound 1. Figure S2: 13C-NMR spectra of compound 1. Figure S3: 13C DEPT-135 NMR spectrum of compound 1. Figure S4: 1H-NMR spectra of compound 2. Figure S5: 13C-NMR spectra of compound 2. Figure S6: 13C DEPT-135 NMR spectrum of compound 2. Figure S7: 1H-NMR spectra of compounds 3 and 4 and the enlarged region used to calculate the relative amount of compounds 3 and 4. Figure S8: 13C-NMR spectra of compounds 3 and 4. Figure S9: 13C DEPT-135 NMR spectrum of compounds 3 and 4. Table S-1. 1H-NMR (400 MHz, CDCl3) and 13C-NMR (101 MHz) of compound 5 and NMR data reported for boehmenan. Figure S10: 1H-NMR spectra of compound 5. Figure S11: 13C-NMR spectra of compound 5. Figure S12: 13C DEPT-135 NMR spectrum of compound 5. Figure S13: 1H-NMR spectra of compounds 6 and 7 and enlarged region used to calculate the relative amount of compounds 6 and 7. Figure S14: 13C-NMR spectra of compounds 6 and 7. Figure S15: 13C DEPT-135 NMR spectrum of compounds 6 and 7. Figure S16 to 19: 2D and 3D interactions of isolated compounds with selected protein targets. [file 3152929.f1.docx]

**Supporting Information**

***In vitro* Antibacterial, DPPH Radical Scavenging Activities, and *In Silico* Molecular Modeling of Isolated Compounds from the Roots of *Clematis hirsuta***

Tolessa Duguma^1^, Yadessa Melaku^1*^, Ankita Garg^1^, Urgessa Ensermu^2^

^1^Department of Applied Chemistry, Adama Science and Technology University, Adama, Ethiopia

^2^Department of Applied Biology, Adama Science and Technology University, Adama, Ethiopia

*Correspondence: yadessamelaku2010@gmail.com

The NMR spectral data used to characterize the structure of isolated compounds (compounds **1**-**7**) are shown in Figure S1-S15. The NMR data of compound **5** is given in Table S-1. The 2D and 3D interactions of isolated compounds with selected targets are shown in Figure S16-19.

 **Figure S1**. ^1^H NMR spectra of compound **1**.

**Figure S2**. ^13^C-NMR spectra of compound **1**.

 **Figure S3**. ^13^C DEPT-135 NMR spectrum of compound **1**.

**Figure S4**. ^1^H-NMR spectra of compound **2**.

**Figure S5**. ^13^C-NMR spectra of compound **2**.

**Figure S6**. ^13^C DEPT-135 NMR spectrum of compound **2**.

**Figure S7**. ^1^H-NMR spectra of compounds **3** and **4** and the enlarged region were used to calculate the relative amount of compounds **3** and **4**.

**Figure S8**. ^13^C-NMR spectra of compounds **3** and **4**.

**Figure S9**. ^13^C DEPT-135 NMR spectrum of compounds **3** and **4**.

Table S-1. ^1^H-NMR (400 MHz, CDCl_3_) and ^13^C-NMR (101 MHz) of compound **5** and NMR data reported for boehmenan

| C $№$ | Compound **5** | | Boehmenan [28] | C $№$ | Compound **5** | | Boehmenan [28] |
| --- | --- | --- | --- | --- | --- | --- | --- |
|  | *δ*^1^H (*J* in Hz) | *δ* ^13^C | *δ* ^13^C/CDCl_3_ |  | *δ*^1^H (*J* in Hz) | *δ* ^13^C | *δ* ^13^C/CDCl_3_ |
| 1 | - | 126.9 | 126.7 | 1’’’ | - | 127.1 | 126.9 |
| 2 | 7.02 (d, *J* = 1.7, 1H) | 109.5 | 109.3 | 2’’’ | 6.89-6.94 (m, 1H) | 109.6 | 109.4 |
| 3 | - | 146.9 | 146.7 | 3’’’ | - | 146.9 | 146.7 |
| 4 | - | 148.1 | 147.9 | 4’’’ | - | 148.3 | 148.1 |
| 5 | 6.86 (d, *J* = 8.3, 1H) | 114.9 | 114.7 | 5’’’ | 6.86 (d, *J* = 8.3, 1H) | 114.9 | 114.7 |
| 6 | 7.06 (dd, *J* = 8.3, 1.7, 1H) | 123.2 | 123.0 | 6’’’ | 6.98 (d, *J* = 1.7, 1H) | 123.2 | 123.1 |
| 7 | 7.47 (d, *J* = 15.9, 1H) | 145.6 | 145.5 | 7’’’ | 7.59 (d, *J* = 15.9. 1H) | 145.0 | 144.9 |
| 8 | 6.23 (d, *J* = 15.9, 1H) | 114.9 | 114.7 | 8’’’ | 6.29 (d, *J* = 15.9, 1H) | 115.6 | 115.4 |
| 9 | - | 167.0 | 167.0 | 9’’’ | - | 167.4 | 167.3 |
| 1’ | - | 132.7 | 132.5 | 3-OCH_3_ | 3.92 (s, 3H) | 56.1-56.2 | 55.9 |
| 2’ | 6.89-6.94 (m, 1H) | 109.0 | 108.8 | 3’-OCH_3_ | 3.84 (s, 3H) |  | 55.9 |
| 3’ | - | 146.8 | 146.6 | 3’’-OCH_3_ | 3.89 (s, 3H) |  | 56.0 |
| 4’ | - | 145.9 | 145.7 | 3’’’OCH_3_ | 3.92 (s, 3H) |  | 55.9 |
| 5’ | 6.91 (d, *J* = 8.1, 1H) | 114.4 | 114.2 | 4-OH | 5.91 (br. s, 1H) | - | - |
| 6’ | 6.89-6.94 (m, 1H) | 119.9 | 119.7 | 4’-OH | 5.64 (br. s, 1H) | - | - |
| 7’ | 5.50 (d, *J*= 7.8, 1H) | 89.1 | 88.9 | 4’’-OH | 5.91 (br. s, 1H) | - | - |
| 8’ | 3.84-3.92 (m, 1H) | 50.9 | 50.7 |  |  |  |  |
| 9’ | 4.41 (dd, *J* = 12.0, 8.0, 1H)  4.58 (dd, *J* =12.0, 4.0, 1H) | 65.6 | 65.4 |  |  |  |  |
| 1’’ | - | 135.1 | 134.9 |  |  |  |  |
| 2’’ | 6.69 (br. s, 1H) | 112.7 | 112.4 |  |  |  |  |
| 3’’ | - | 144.3 | 144.1 |  |  |  |  |
| 4’’ | - | 146.4 | 146.2 |  |  |  |  |
| 5’’ | - | 127.6 | 127.4 |  |  |  |  |
| 6’’ | 6.71 (br. s, 1H) | 116.3 | 116.1 |  |  |  |  |
| 7’’ | 2.70 (t, *J* = 7.8, 2H) | 32.3 | 32.1 |  |  |  |  |
| 8’’ | 2.00-2.05 (m, 2H) | 30.9 | 30.7 |  |  |  |  |
| 9’’ | 4.23 (t, *J* = 6.5, 2H) | 63.9 | 63.7 |  |  |  |  |

**Figure S10**. ^1^H-NMR spectra of compound **5**.

**Figure S11**. ^13^C-NMR spectra of compound **5**.

**Figure S12**. ^13^C DEPT-135 NMR spectrum of compound **5**.

 **Figure S13**. ^1^H-NMR spectra of compounds **6** and **7** and enlarged region were used to calculate the relative amount of compounds **6** and **7**.

**Figure S14**. ^13^C-NMR spectra of compounds **6** and **7**.

**Figure S15**. ^13^C DEPT-135 NMR spectrum of compounds **6** and **7**.

| Target | Comp | 3D | 2D |
| --- | --- | --- | --- |
| 7P2M | **3** | 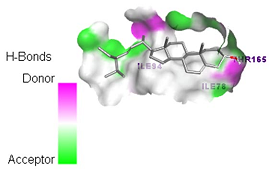 | 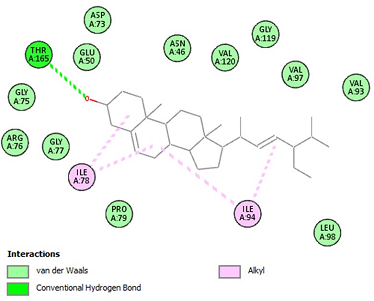 |
|  | **4** | 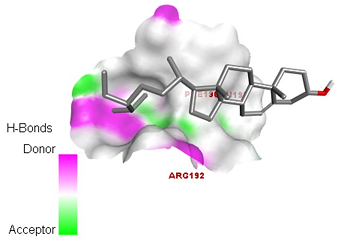 | 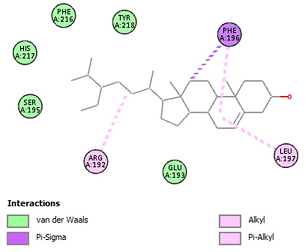 |
|  | **5** | 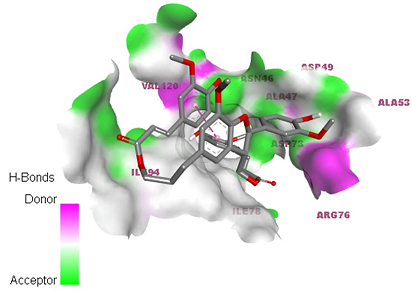 | 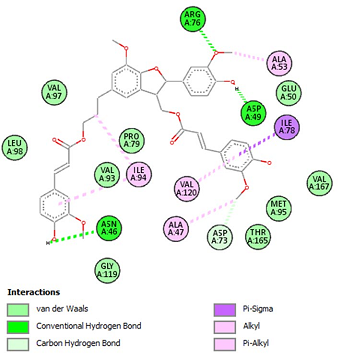 |

**Figure S16**. The 3D and 2D binding interactions of compound **3-7** and ciprofloxacin against *E*. *coli* DNA Gyrase B (PDB ID: 7P2M).

| Target | Comp | 3D | 2D |
| --- | --- | --- | --- |
| 7P2M | **6** | 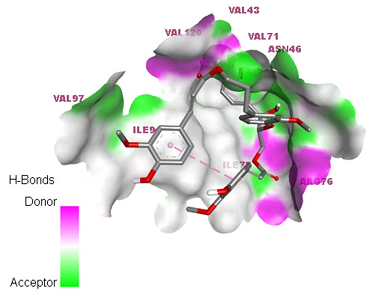 | 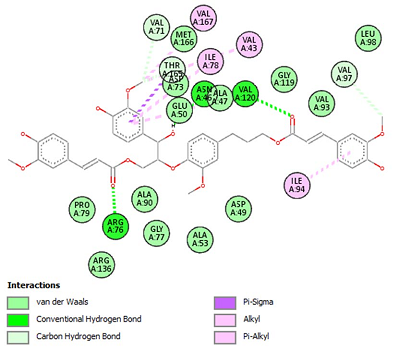 |
|  | **7** | 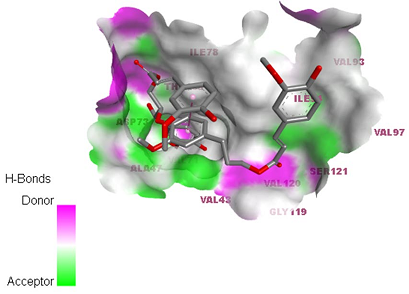 | 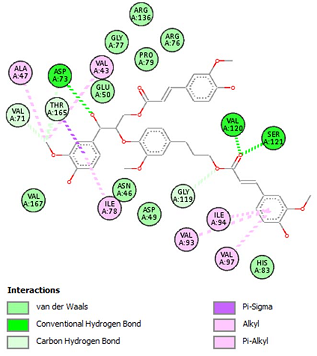 |
|  | Ciprofloxacin | 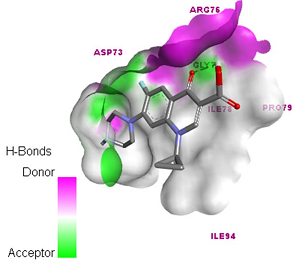 | 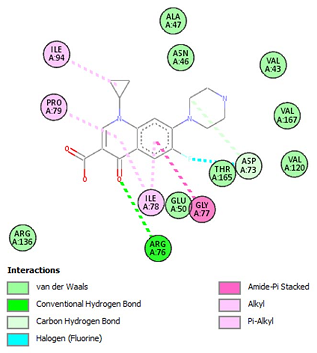 |

**Figure S16**. Cont’d

| Target | Comp | 3D | 2D |
| --- | --- | --- | --- |
| 5OE4 | **4** | 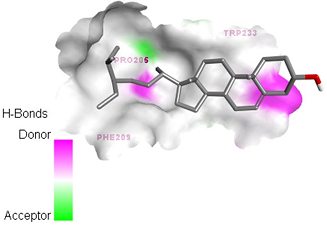 | 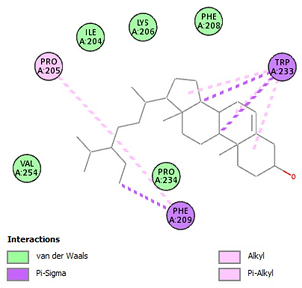 |
|  | **5** | 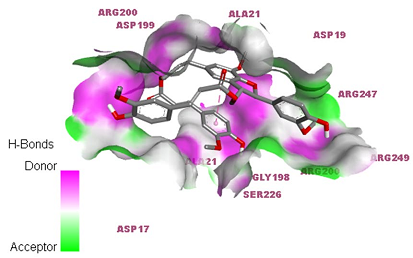 | 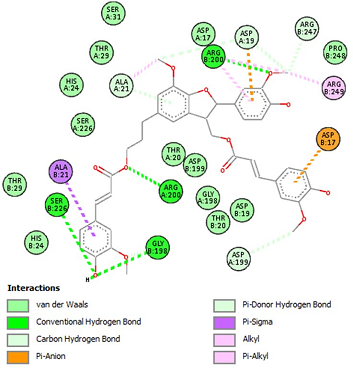 |
|  | **6** | 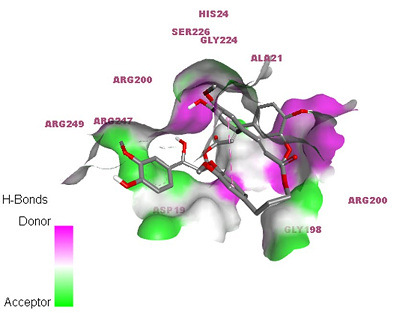 | 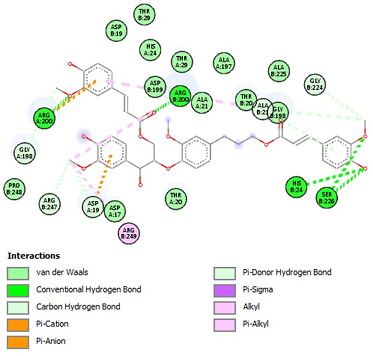 |
|  | **7** | 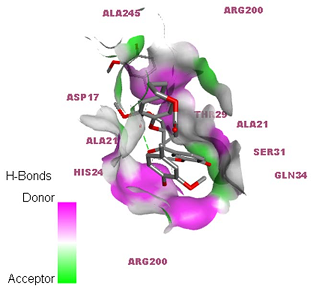 | 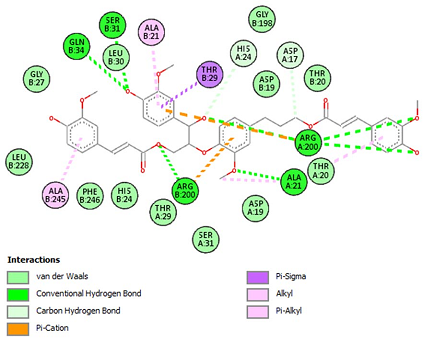 |

**Figure S17**. The 3D and 2D binding interactions of compound **4-7** against *Pseudomonas* quinolone signal A, PqsA,(PDB ID: 5OE4).

| Target | Comp | 3D | 2D |
| --- | --- | --- | --- |
| 4G1N | **3** | 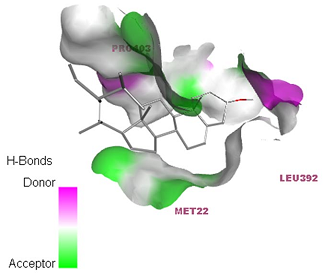 | 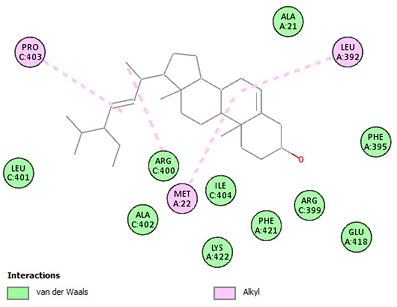 |
|  | **4** | 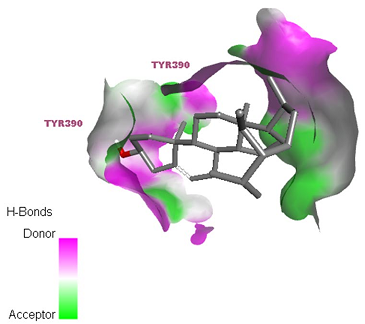 | 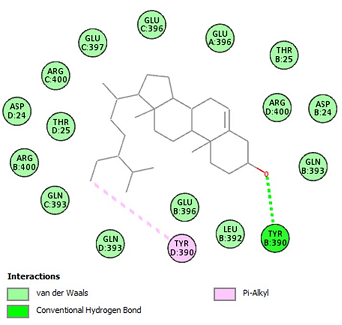 |
|  | **6** | 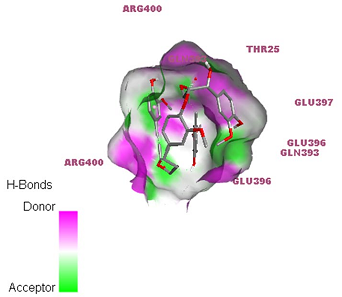 | 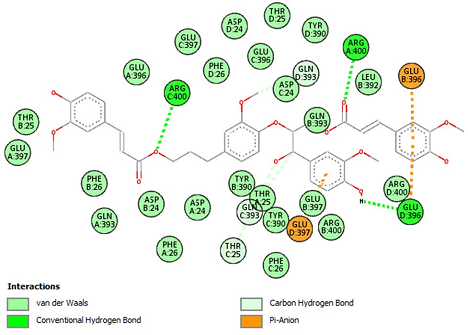 |
|  | **7** | 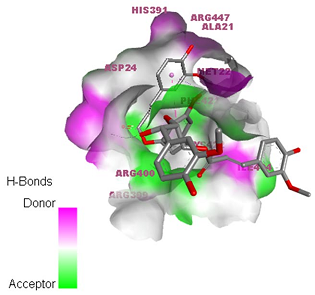 | 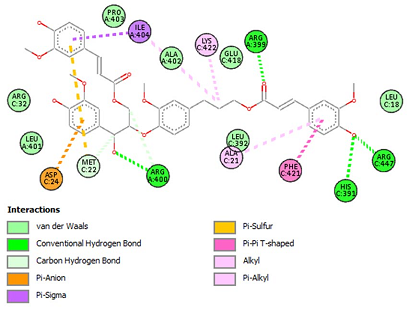 |

**Figure S18.** The 3D and 2D binding interactions of compounds **3**, **4**, **6**, and **7** against Pyruvate kinase M2, PKM2, (PDB ID: 4G1N).

| Target | Comp | 3D | 2D |
| --- | --- | --- | --- |
| 3QX3 | **3** | 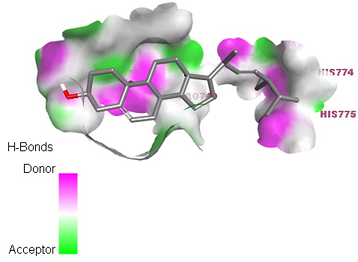 | 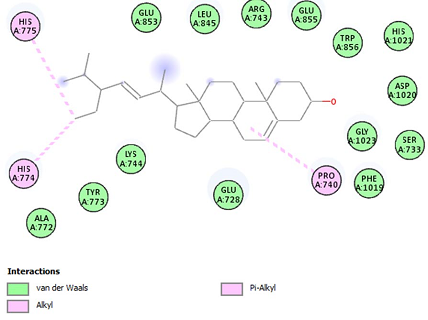 |
|  | **4** | 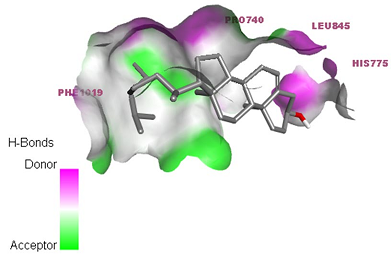 | 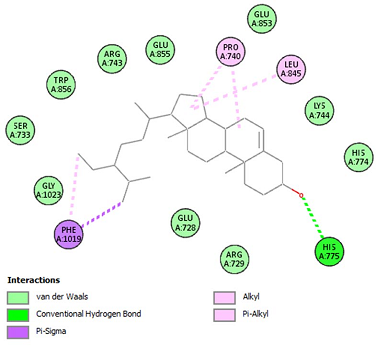 |
|  | **5** | 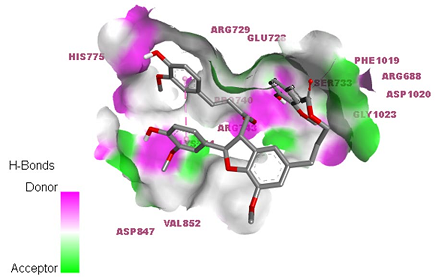 | 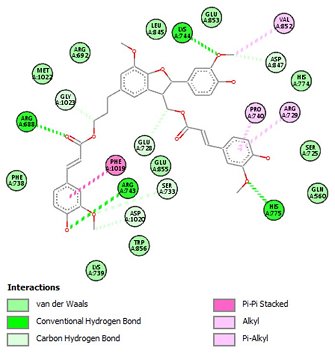 |

**Figure S19**. The 3D and 2D binding interactions of compound **3-7** and abiraterone against human topoisomerase *IIβ* (PDB ID: 3QX3).

| Target | Comp | 3D | 2D |
| --- | --- | --- | --- |
| 3QX3 | **6** | 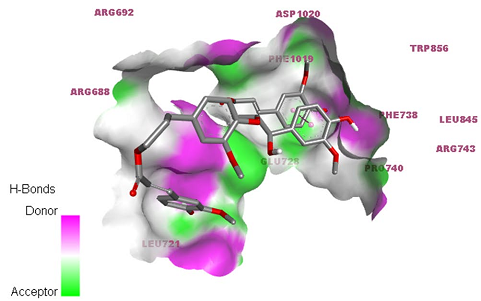 | 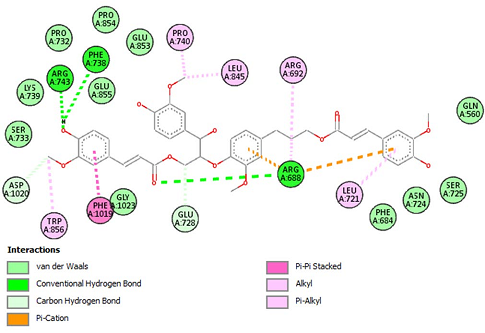 |
|  | **7** | 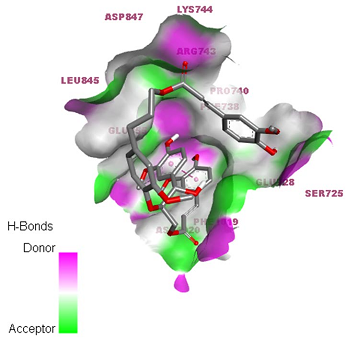 | 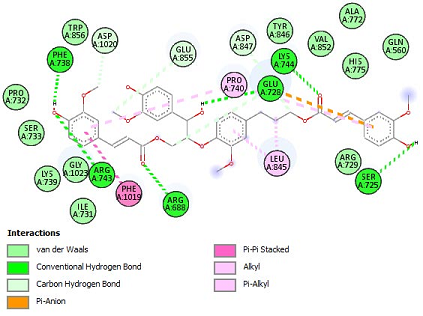 |
|  | Abiraterone | 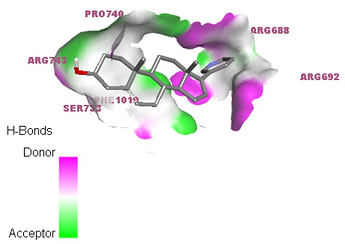 | 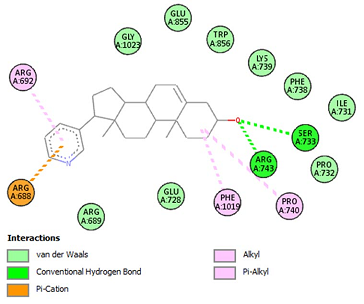 |

**Figure S19**. Cont’d
